# Evaluation of 7 Tesla pTx Body Coils Regarding Transmit and Receive Performance

**DOI:** 10.1002/mrm.70557

**Published:** 2026-08-14

**Authors:** Johannes A. Grimm, Oliver Kraff, Markus W. May, Max Lutz, Stephan Orzada, Thomas M. Fiedler, Steen Moeller, Simon Schmidt, Harald H. Quick, Armin M. Nagel, Mark E. Ladd, Sebastian Schmitter

**Affiliations:** ^1^ Medical Physics in Radiology, German Cancer Research Center (DKFZ) Heidelberg Germany; ^2^ Faculty of Physics and Astronomy Heidelberg University Heidelberg Germany; ^3^ Erwin L. Hahn Institute for Magnetic Resonance Imaging University Duisburg‐Essen Essen Germany; ^4^ High‐Field and Hybrid MR Imaging University Hospital Essen Essen Germany; ^5^ Physikalisch‐Technische Bundesanstalt (PTB) Braunschweig Germany; ^6^ Center for Magnetic Resonance Research University of Minnesota Minneapolis Minnesota USA; ^7^ Institute of Radiology, University Hospital Erlangen Friedrich‐Alexander‐Universität Erlangen‐Nürnberg (FAU) Nürnberg Germany

**Keywords:** 7T, body MR imaging, pTx, radiofrequency (RF) coil comparison, SNR, ultra‐high field

## Abstract

**Purpose:**

Most sites develop and use custom coils for 7 T body MRI since no standard pTx body coil exists, leading to diverse coil designs differing in transmit element type, layout, and number of receive channels. This study investigates and compares eight existing coils, including one remote body coil array and seven local arrays, regarding their transmit and receive performance.

**Methods:**

Phantom measurements were conducted with all coils on the same 7 T scanner, using the same phantom and imaging protocol. Transmit performance was compared in terms of *B*_1_^+^ efficiency and coverage. Receive performance was compared in terms of SNR, coverage, noise correlation, and g‐factors.

**Results:**

Mean *B*_1_^+^ efficiency ranged from 2.02 to 4.33 μT/√kW across configurations, lowest for the remote array and highest for an 8Tx8Rx local array. Central SNR ranged from 278 to 1072, increasing with receive element count and peaking for an 8Tx32Rx configuration. HF‐excitation‐coverage ranged from 34 to 384 mm, and HF‐receive‐coverage from 135 to 384 mm, with the remote array combined with a local 32Rx array achieving highest coverage. Acceleration performance improved with increasing receive element count in the corresponding direction. An 8Tx16Rx and an 8Tx32Rx arrays performed best in LR‐/AP‐direction and the 32Rx array in HF‐direction.

**Conclusion:**

No existing local pTx coil provides universally optimal performance, as each design offers different advantages in either *B*_1_^+^ efficiency, coverage, SNR, or acceleration. Among the local arrays, the 8Tx32Rx array (C5) might represent a reasonable compromise with respect to the parameters evaluated in this study, as it exhibits the highest central SNR, high coverage, and acceleration.

## 1 Introduction

Ultra‐high field (UHF) MRI at 7 T can improve image quality compared to clinical field strengths at 1.5 T and 3 T through improvements in SNR and contrast‐to‐noise ratio [1]. However, due to the shortened RF wavelength at 7 T, *B*_1_^+^ field inhomogeneities occur, which can lead to flip angle (FA) variations and dropouts, posing challenges particularly in body imaging applications.

For UHF brain imaging at 7 T, two commercially available head coils, one with 1 transmit (Tx)/32 receive (Rx) elements and one enabling parallel transmission (pTx) with 8 Tx/32 Rx elements (both Nova Medical Inc., Wilmington, USA) are available and have been used at many 7 T sites. This availability has greatly facilitated multi‐center studies and the dissemination of standardized brain imaging protocols [2, 3, 4, 5].

In contrast to the brain, multi‐transmit‐channel coils can be considered mandatory for most body applications at UHF due to the more inhomogeneous *B*_1_^+^ profiles in the human body, together with the need to shape the electric field to limit the local specific absorption rate (SAR). To address both challenges, pTx methods [6] have been developed that enable spatial and/or temporal modulation of the transmit fields by utilizing local RF coil arrays with multiple transmit elements that are independently driven by separate transmit channels [7]. The number, the type and the arrangement of the elements remain an active topig of research, for example, for applications in the human brain [8, 9, 10]. When combined with such multi‐channel transmit hardware, pTx methods can mitigate *B*_1_^+^ inhomogeneities and achieve improved homogeneity or increased *B*_1_^+^ efficiency in the head [11, 12] as well as in different body regions, that is, prostate [13], heart [14], liver [15], or whole abdomen [16].

In contrast to the human brain, however, there is no standardized body coil available for UHF body MR imaging despite a growing interest in body applications in recent years [17, 18]. Thus, most research groups and institutions have either purchased local transmit/receive prototype body coils from different vendors or have independently developed their own RF arrays over the past decades [19, 20], resulting in a wide variety of coil designs with differences in transmit element type, layout, and number of receive channels.

A standardized pTx body array would offer numerous benefits: it would enable 7T body MRI also at sites without in‐house RF engineering expertise, support the dissemination of imaging protocols, sequences, and techniques across institutions, and provide a foundation for multi‐center studies. Moreover, such standardization would likely accelerate the extension of current CE/FDA approvals of 7T systems toward full‐body applications by facilitating the demonstration of 7 T advantages in the torso across sites. Thus, we initiated a study to systematically compare presently existing pTx body arrays at different UHF sites in terms of their transmit and receive performance.

Specifically, eight existing RF arrays were evaluated, resulting in eight different transmit/receive configurations, including five in‐house developed coils and three vendor‐provided coils, originating from four different institutions in Germany. These configurations differ in design, element type (loops, dipoles, meander striplines (MS)), housing, and number of transmit and receive channels (Table 1). This study provides a comprehensive comparison of both transmit and receive performance, including *B*_1_^+^ efficiency, field of excitation (FoX) in head‐foot (HF) direction, SNR analysis, noise correlation, and parallel imaging capability.

**TABLE 1:** Characterization of the pTx coils used in this study. MS: Meander stripline; L: Loops; D: Dipoles; The coils are from the following sites: C1 (DKFZ): 32Tx32Rx, C2 (DKFZ): 8Tx8Rx, C3 (FAU): 8Tx16Rx, C4 (ELH): 8Tx8Rx, C5 (ELH): 8Tx32Rx, C6 (PTB): 8Tx32Rx, C7 (PTB): 8Tx16Rx, C8 (DKFZ): 32Rx.

	C1	C2	C3	C4	C5	C6	C7	C8
Coil type	Remote	Local	Local	Local	Local	Local	Local	Local
Tx type	MS	MS	L	MS	MS	D + L	D/L	—
Rx type	MS	MS	L	MS	MS + L	D + L	D + L	L
# Tx/Rx elements	32/32	8/8	8/16	8/8	8/32	8/32	8/16	−/32
Site	DKFZ, Heidelberg	DKFZ, Heidelberg	FAU, Erlangen	ELH, Essen	ELH, Essen	PTB, Berlin	PTB, Berlin	DKFZ, Heidelberg

All coils have been driven with an 8‐channel Siemens pTx system except for coils C1 and C8, which used the Siemens single‐channel system in combination with a self‐built 32 × 2 kW pTx system [21].

## 2 Methods

### 2.1 Hardware

All measurements were performed on the same 7 T system (MAGNETOM 7 T, Siemens Healthineers AG, Germany), which is equipped with an 8 × 1 kW pTx system. A total of eight different RF arrays, denoted as C1 to C8 in the following, originating from four different German research sites, including one remote body coil array and seven local arrays, were evaluated (c.f. Table 1). These arrays were investigated in 8 unique transmit configurations (C1‐C6, C7 dipoles and C7 loops) and 8 receive configurations (C1‐C6, C7 transmitting with dipoles only and receiving with both, dipoles and loops and C8) and summarized below. Further details of the coil design and elements are presented in Figure S1.

- **C1:** The first custom‐built array consists of a remote body coil array mounted on the outside of the bore liner, which is a setup similar to a conventional RF body coil used in clinical systems at lower field strengths. It features 32 MS elements, which can be used for both transmit (Tx) and receive (Rx) [21].
- **C2:** A custom‐built replica based on the coil by Orzada et al. [22], incorporating eight MS transmit/receive elements. It is designed as a local coil for anterior and posterior placement around the body.
- **C3:** A commercial RF array (Rapid Biomedical GmbH, Rimpar, Germany) consisting of 16 loop elements [23] with a modified coil housing to ensure compatibility with a large ^23^Na volume resonator for combined ^23^Na/^1^H imaging [24]. Each of the anterior and posterior parts of the array includes a central circular loop surrounded by seven trapezoidal‐shaped loops arranged in a heptagonal geometry [25]. For transmission, two neighboring loop elements are combined using a fixed hardware shim to form eight transmit channels.
- **C4:** A custom‐built array based on the same design as C2, but the posterior housing is modified with a slight curvature to improve anatomical conformity to the abdomen [26]. This configuration places the elements up to 5 cm closer to the phantom compared to a flat posterior array.
- **C5:** A custom‐built array which includes eight MS elements used for both transmit and receive and additionally integrates 24 receive‐only loop elements positioned between the MS elements and the array casing [27].
- **C6:** A commercial 32‐element body array (MRI.TOOLS GmbH, Berlin, Germany), which incorporates eight dipole elements, each paired with three overlapping loop elements located along the dipole [28]. For transmission, each dipole and its associated loops are combined using a hardware‐fixed phase configuration.
- **C7:** A custom‐built array based on a design from Ertürk et al. [29] with a modified coil housing [30]. This array features eight transmission blocks, each consisting of one loop and one dipole located on top of the loop. It was evaluated in two transmit configurations, as the original coil was intended to be used with a 16‐channel pTx system: excitation using only the dipole elements (C7 dipoles) and excitation using only the loop elements (C7 loops). In both cases, all 16 elements were used for reception.
- **C8:** A commercial receive‐only array (Rapid Biomedical GmbH, Rimpar, Germany) consisting of 32 loop elements. This coil was used for reception in combination with the remote array C1 for transmission.

Coils C2, C4, C5, and C7 were operated using the same transmit/receive‐switch and preamplifier box, whereas the remaining coils were each driven by their respective dedicated transmit/receive‐switch and preamplifier units.

### 2.2 Measurements

Each coil was centered on the identical body phantom (see Figure 1) (length = 51 cm, height = 23 cm, diameter top = 12 cm, diameter center = 35 cm), filled with a solution of 49% polyvinylpyrrolidone (PVP), 1.8% sodium chloride, and 49.2% water to mimic the dielectric properties of tissue of the human body (Figure S2). The phantom's center was aligned with the scanner's isocenter. Dielectric properties were characterized using a DAK probe (Schmid & Partner Engineering AG, Zürich, Switzerland). Measurements yielded a relative permittivity (ε) of 36 and an electrical conductivity (σ) of 0.45 S/m. A transmit reference voltage of 170 V per channel was used for all local transmit/receive coils (C2‐C7), and 350 V was used for C1 and C8. After second‐order *B*_0_‐shimming over the entire volume was performed, transverse 2D multi‐slice relative *B*_1_^+^ (B1R) maps [31] were acquired (Figure S3). For each scan, a circular region of interest (ROI) with a diameter of 6 cm was selected using a semi‐automatic procedure (c.f. Figure 2), ensuring that the same position was used for optimization across all eight transmit configurations.

**FIGURE 1:**
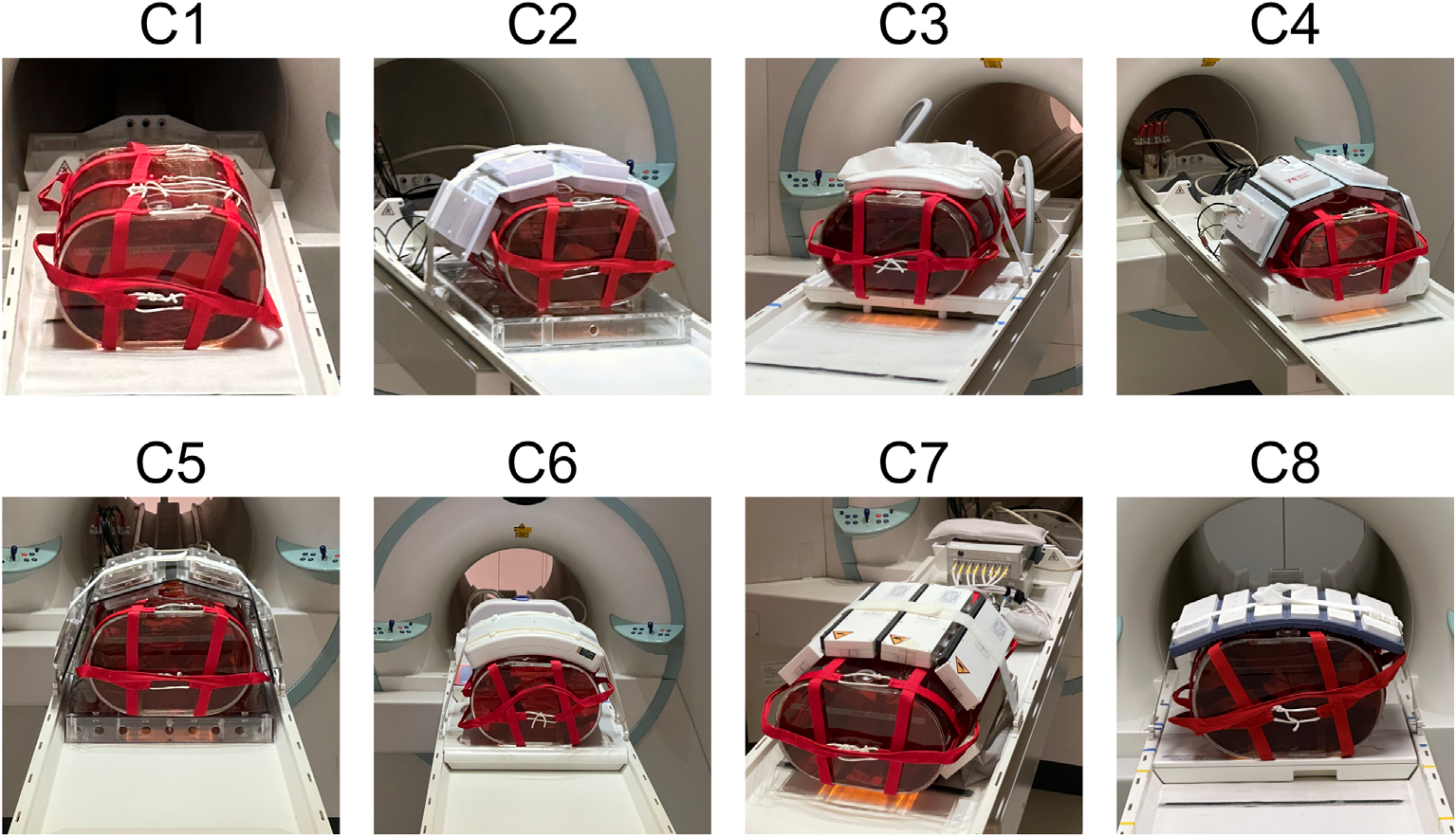
Measurement setup shown for all 8 coils. C1: 32Tx32Rx, C2: 8Tx8Rx, C3: 8Tx16Rx, C4: 8Tx8Rx, C5: 8Tx32Rx, C6: 8Tx32Rx, C7: 8Tx16Rx, C8: 32Rx.

**FIGURE 2:**
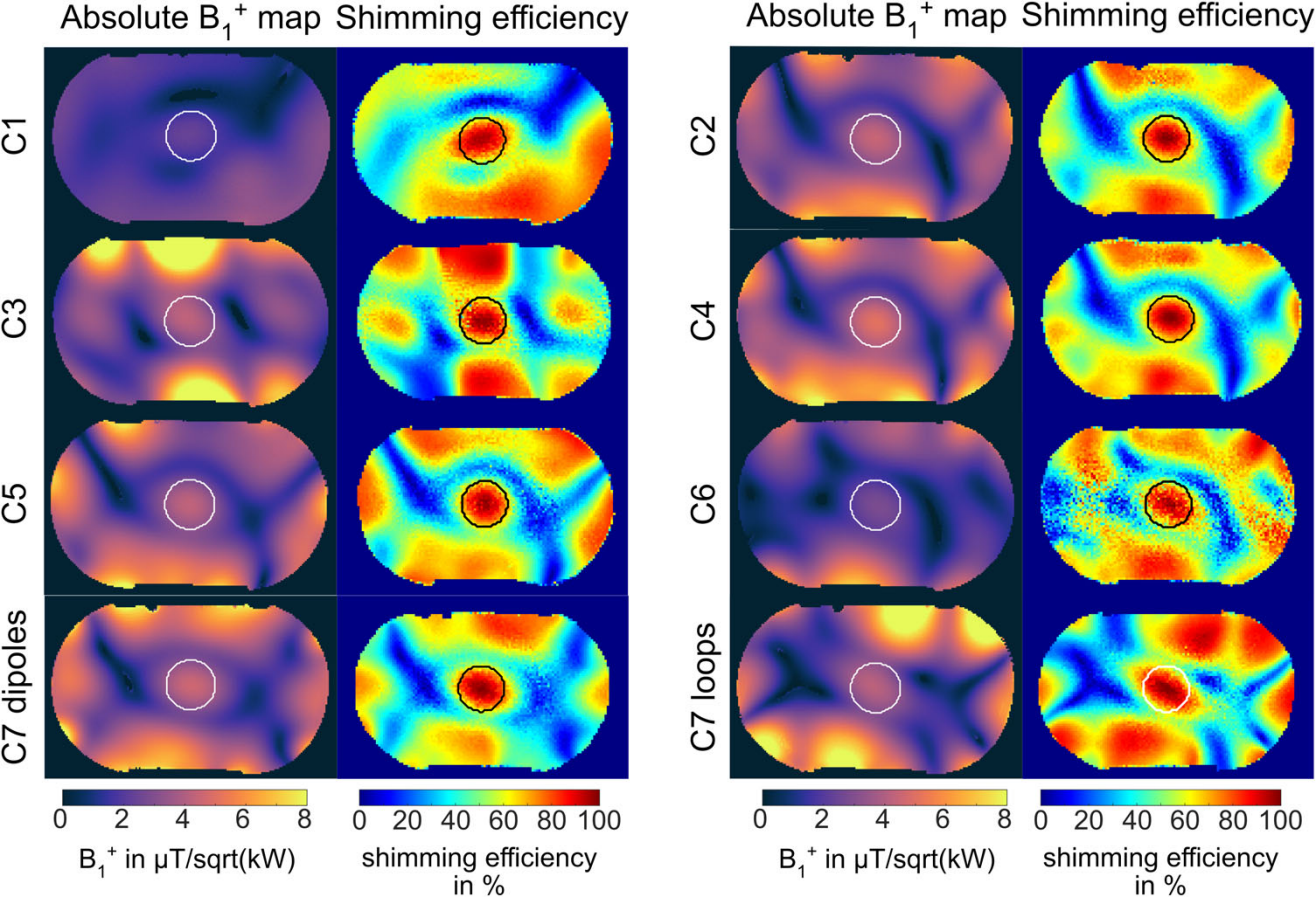
Absolute *B*_1_^+^ and shimming efficiency maps of an efficiency shim for all transmit configurations. C1: 32Tx32Rx, C2: 8Tx8Rx, C3: 8Tx16Rx, C4: 8Tx8Rx, C5: 8Tx32Rx, C6: 8Tx32Rx, C7 dipoles: 8Tx16Rx using dipoles as transmit, C7 loops: 8Tx16Rx using loops for transmit.

Phase‐only *B*_1_^+^ shimming was performed on the isocenter slice to maximize the *B*_1_^+^ efficiency η [13, 28], that is, $\max\left(\eta \right)=\max\left(\frac{\left|\sum_{i}^{N_{\mathrm{ch}}}B_{1,i}^{+}b_{i}\right|}{\sum_{i}^{N_{\mathrm{ch}}}\left|B_{1,i}^{+}\right|}\right)$, with $N_{\mathrm{ch}}$ the number of channels, $B_{1,i}^{+}$ the complex $B_{1}^{+}$ field of the i‐th channel and $b_{i}$ the shim of the i‐th channel, within the ROI using MATLAB. All subsequent scans were acquired with (i) zero‐phase mode, that is, no additional phases applied to the respective coil elements, (ii) CP+ mode, (iii) CP2+ mode, and (iv) the efficiency shim. Absolute 2D *B*_1_^+^ maps were acquired in all three orientations at the isocenter using an MRF‐based *B*_1_^+^‐mapping sequence (Figure S3) [32, 33]. Additionally a GRE image (GRE‐SNR) was acquired in the center transversal slice (Figure S3).

#### 2.2.1 Transmit Performance Analysis

*B*_1_^+^ efficiency of the transmit configurations was evaluated within the ROI by normalizing the absolute *B*_1_^+^ maps to the input RF power of the RF amplifiers at the patient table head (directional Couplers for C1). In addition, the shimming efficiency η was also assessed within the same ROI.

*B*_1,max_^+^ along the HF‐direction was derived from the B1R maps as follows (see Figure S4):

$$B_{1,\max}^{+}=\mathrm{SOM}\left(B1R\right)*\text{mean}\left(B_{1,\mathrm{eff}}^{+}\;\right)*\sqrt{1.2\mathrm{kW}*N_{\mathrm{ch}}}$$

Here, SOM(B1R) denotes the sum of magnitudes of the 3D B1R dataset along the transmit channels normalized to the center sagittal/coronal line. This corresponds to 100% constructive interference of all channels. $\text{Mean}\left(B_{1,\mathrm{eff}}^{+}\;\right)$ denotes the mean *B*_1_^+^ efficiency value in $\frac{\mu T}{\sqrt{\mathrm{kW}}}$ of the coil within the central ROI of the transversal slice. An available input power of 1.2kW per channel was assumed at the patient table head for C2‐C7 and at the directional couplers for C1, which corresponds to an amplifier output power of 2 kW per channel. This resulted in a total transmitted power of P_total_ = 64 kW for C1 and P_total_ = 16 kW for C2‐C7.

The field of excitation (FoX) was defined as the region where the *B*_1_,max^+^ exceeded 7.8μT, which corresponds to the *B*_1_^+^ value that is required to generate a rectangular‐shaped 180° pulse in 1500 μs.

S‐parameter measurements for all local coils were performed on the workbench using the phantom.

#### 2.2.2 Receive Performance Analysis

SNR maps for shim (ii–iv) and for all receive configurations were computed in the central transversal slice from the GRE‐SNR images according to the method of Kellman et al. [34] These maps were subsequently corrected for *B*_1_^+^ variations using FA maps obtained from the B1‐MRF sequence and for receive bandwidth ($BW_{R}$) and voxel volume V [35, 36]:

$$\mathrm{SN}R_{\text{corrected}}\left(x,y\right)=\mathrm{SNR}\left(x,y\right)*\frac{\sqrt{BW_{R}}}{V*\sin\left(\mathrm{FA}\left(x,y\right)\right)},$$

with x,y the pixel position.

The corrected SNR maps for each shim (ii–iv) were then combined following the approach of Brunheim et al. [37] resulting in a single FA corrected SNR map per receive configuration.

SNR maps in the central sagittal and coronal slices were derived from the GRE images acquired as part of the B1R sequence. For these, the channel‐wise GRE images were combined using zero‐phase shim as well as the CP+ and CP2+ modes, and then post‐processed in the same way as the transverse SNR maps. To account for protocol differences between the GRE‐SNR and the GRE acquisitions used for the B1R (GRE‐B1R), the sagittal and coronal SNR maps were scaled by the ratio of the central SNR value obtained from the transverse GRE‐SNR map to that from the transverse GRE‐B1R‐based SNR maps.

To evaluate inter‐element coupling, noise correlation matrices were computed from noise covariance matrices using MATLAB's internal correlation function.

Parallel imaging performance was analyzed using g‐factor maps for acceleration factors of *R* = 2 to *R* = 5 in all three orientations. For anterior–posterior (AP) and left–right (LR) direction, sensitivity maps were derived from the GRE images acquired with the CP+ mode. For HF‐direction, sensitivity maps were derived from the image data of the first GRE‐based B1R acquisition, which transmits on all channels using the zero phase mode. Coil sensitivity maps were determined using the method of Walsh et al. [38] G‐factor maps for each orientation were computed analytically for the SENSE technique [39, 40].

For C7, the receive performance was evaluated using measurements acquired with the dipoles in transmit mode.

## 3 Results

### 3.1 Transmit Performance Analysis

#### 3.1.1 *B*_1_^+^ Efficiency

Figure 2 displays the absolute *B*_1_^+^ efficiency maps alongside the shimming efficiency maps derived from the optimized efficiency shim. The corresponding mean, standard deviation, and maximum *B*_1_^+^ values, as well as shimming efficiency values within the central ROI, are summarized in Figure 3.

**FIGURE 3:**
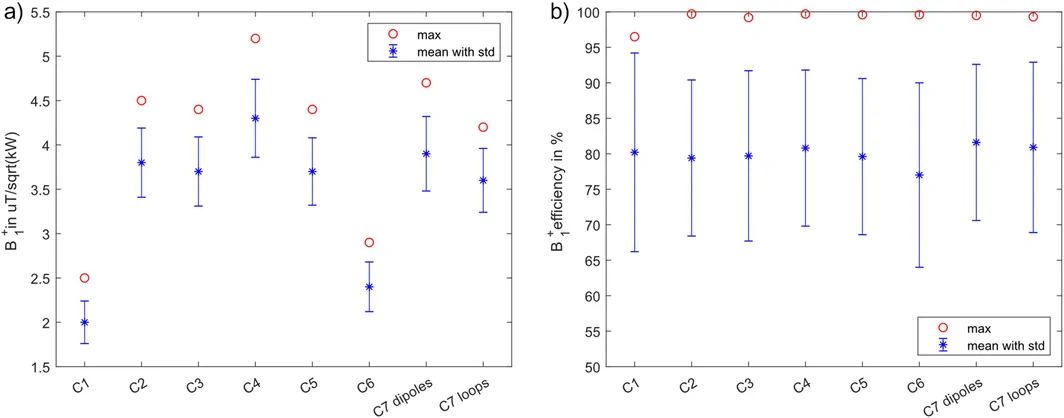
(a) mean, std. and max *B*_1_^+^ values and (b) mean, std. and max shimming efficiency values within the circular ROI in the center of the phantom for all transmit configurations. C1: 32Tx32Rx, C2: 8Tx8Rx, C3: 8Tx16Rx, C4: 8Tx8Rx, C5: 8Tx32Rx, C6: 8Tx32Rx, C7 dipoles: 8Tx16Rx using dipoles as transmit, C7 loops: 8Tx16Rx using loops for transmit.

All local coil arrays achieved maximum shimming efficiency values close to 100% within the ROI in the phantom center, with mean efficiency values ranging between 79% and 82% across the ROI. The mean *B*_1_^+^ efficiency value across the ROI for the local transmit configurations ranged from 2.4 μT/√kW to 4.3 μT/√kW and the maximum *B*_1_^+^ efficiency values ranged from 2.9 μT/√kW to 5.2 μT/√kW, with C4 exhibiting the highest mean and maximum *B*_1_^+^ efficiency values.

Among the local arrays, the 8Tx32Rx coil C6 yielded the lowest mean and maximum *B*_1_^+^ efficiency values within the ROI, with 2.4 μT/√kW and 2.9 μT/√kW, respectively. In comparison, the 8Tx32Rx array C5 with identical numbers of transmit and receive channels showed an increase in mean *B*_1_^+^ efficiency of 54%.

When comparing the 8Tx32Rx array C5 with the 8Tx8Rx array C2, which has a similar layout of posterior and anterior transmit elements but lacks the additional loop receive elements, both arrays achieved comparable *B*_1_^+^ efficiency values and shimming efficiency within the ROI. In contrast, C4, which also uses the same element type but in a modified housing with anatomically conformed element positioning in the posterior part, achieved a 13% increase in *B*_1_^+^ efficiency.

The 8Tx16Rx coil C7 demonstrated an 8% increase in mean *B*_1_^+^ efficiency when using the dipole elements for transmission (C7 dipoles) compared to the loop‐based transmit configuration (C7 loops). Furthermore, C7 dipoles achieved a mean *B*_1_^+^ efficiency value similar to the MS transmit arrays (C2, C4), indicating comparable performance between dipole and MS element designs.

In contrast, the 8Tx16Rx coil C3, which uses loop elements for transmission, produced lower *B*_1_^+^ efficiency values in the ROI compared to both the MS‐based arrays (C2, C4) and the dipole‐based configuration (C7 dipoles).

The remote 32Tx/32Rx body array C1 showed the lowest *B*_1_^+^ efficiency values overall, with mean and maximum *B*_1_^+^ values of 2.0 μT/√kW and 2.5 μT/√kW, respectively, despite achieving a similarly high shimming efficiency (mean/max: 80.2%/96.5%). This reduced performance can be attributed to the coil's location outside the bore liner, thus with a larger distance to the phantom and the large excitation coverage.

#### 3.1.2 Field of Excitation

Figure 4 presents the normalized 2D excitation profile SOM(B1R) in the central sagittal and central coronal slice. The corresponding *B*_1,max_^+^ profiles along the isocenter line in the HF‐direction for all transmit configurations are shown in Figure 5, and the corresponding FoX is summarized in the figure caption of Figure 5.

**FIGURE 4:**
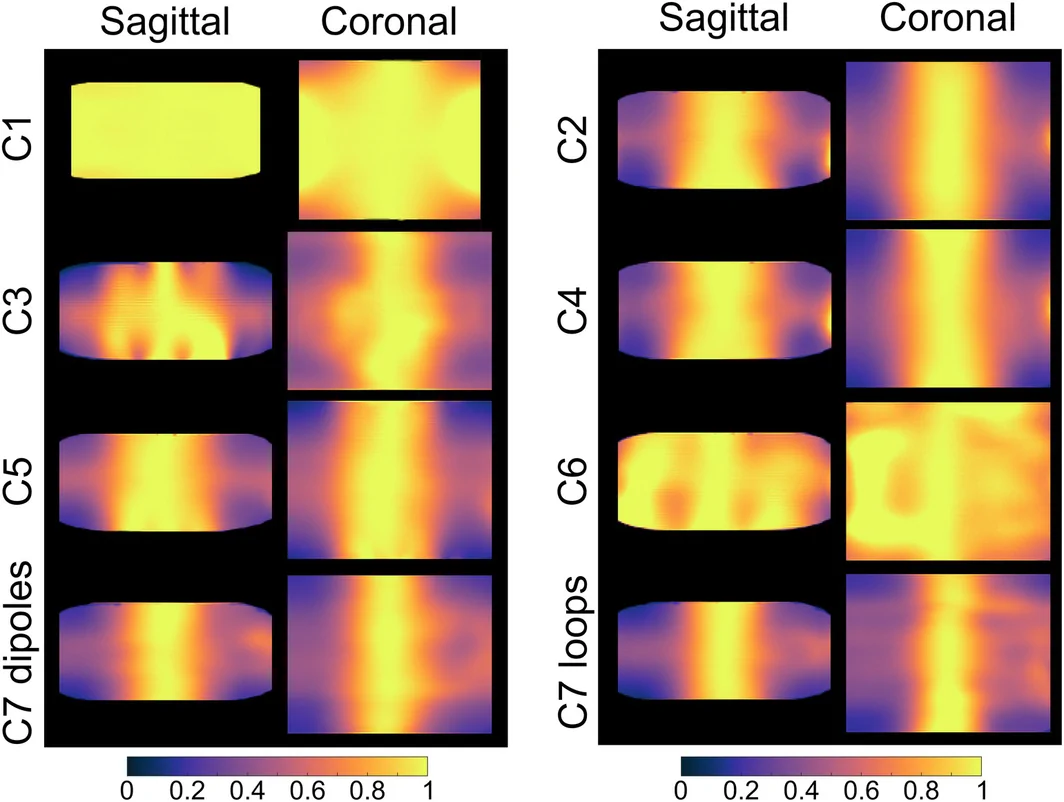
Sagittal and coronal normalized SOM B1R images normalized to the center line for all transmit configurations. C1: 32Tx32Rx, C2: 8Tx8Rx, C3: 8Tx16Rx, C4: 8Tx8Rx, C5: 8Tx32Rx, C6: 8Tx32Rx, C7 dipoles: 8Tx16Rx using dipoles as transmit, C7 loops: 8Tx16Rx using loops for transmit.

**FIGURE 5:**
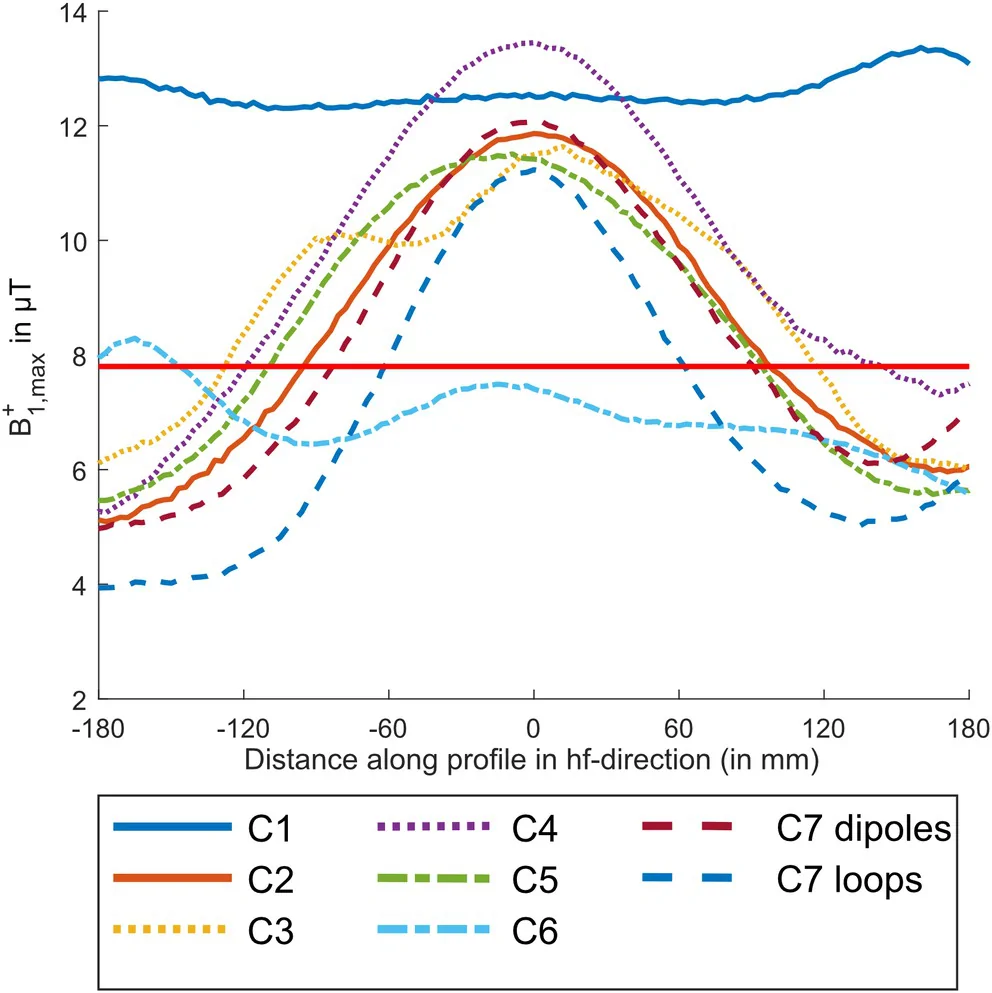
HF‐excitation profile for all transmit configurations along the isocenter line. The horizontal red line marks the excitation width criterion, where *B*_1_,max^+^ exceeds 8.7 μT. The FoX of the transmit configuration is given by: C1 = 384 mm, C2 = 195 mm, C3 = 246 mm, C4 = 264 mm, C5 = 207 mm, C6 34 mm, C7 dipoles = 174 mm, C7 loops = 126 mm.

Among the local transmit configurations, C6 yielded the lowest *B*_1_^+^ efficiency, approximately 30% lower than that of the other local arrays and it consequently did not reach the required *B*_1_^+^ of 7.8 μT needed to achieve a 180° pulse within 1.5 ms. However, C6 exhibited the least variation in the *B*_1,max_^+^ profile along the HF‐direction, due to the distribution of its multiple transmit elements in that orientation.

In contrast, C3, another coil that has elements partially arranged along the HF‐direction due to the circular locations of the elements, achieved the second largest HF‐FoX among the local arrays. However, the 2D sagittal excitation profiles revealed larger *B*_1_^+^ variations in HF‐direction closer to the elements as compared to the center line.

For the local transmit configurations with transmit elements arranged in a single ring (C2, C4, C5, C7) the 2D sagittal and coronal excitation profiles (Figure 4) revealed that the maximum excitation is achieved at the isocenter of the elements along the HF‐direction, with a rapid signal decrease toward the edges of the phantom. This trend is also reflected in the profile plots of the isocenter line (Figure 5).

The highest FoX among the local transmit arrays is achieved by C4, which at the same time achieved the highest *B*_1_^+^ efficiency.

The C7 configuration using loop elements for transmit (C7 loops) achieved the lowest FoX among all arrays with one transmit element in HF‐direction, showing a 35%–52% reduction compared to MS element coils. However, substituting the loop elements with dipoles in the C7 configuration (C7 dipoles) led to a 38% improvement in FoX, yielding the second‐highest *B*_1_^+^ efficiency of all local transmit configurations.

Focusing on the local transmit configurations employing MS elements (C2, C4, C5), the C4 array provided the highest FoX in this approach, outperforming the other two by 28% to 35%.

The remote array (C1) exhibited the most uniform excitation profile, with *B*_1_^+^ variations along the HF‐direction remaining below 11% in sagittal orientation. It achieved a minimum *B*_1_^+^ of 12.3 μT, thereby exceeding the excitation threshold of 7.8 μT across the entire phantom. Additionally, C1 provided the highest *B*_1_^+^ values throughout the phantom. This superior performance is attributed to its configuration of 32 transmit elements, enabling four times the total available RF power compared to local arrays equipped with only eight transmit elements.

A general trend observed across the local transmit configurations was the presence of higher *B*_1_,_max_^+^ values toward the head side compared to the foot side. In several cases, the *B*_1_,_max_^+^ even increased near the edge of the phantom toward the head. This asymmetry is likely due to the influence of the transmit/receive cables, which connect the coil elements to the switching hardware and can locally perturb the *B*_1_^+^ field distribution as well as due to fields penetrating the phantom from both ends.

#### 3.1.3 S‐Parameters

Figure 6a shows the S‐parameters of all local coils measured in the phantom. Across these coils, S_ii_ parameters ranged from −6.7 dB to −27.3 dB, while S_ij_ parameters ranged from −13.8 dB to −66 dB.

**FIGURE 6:**
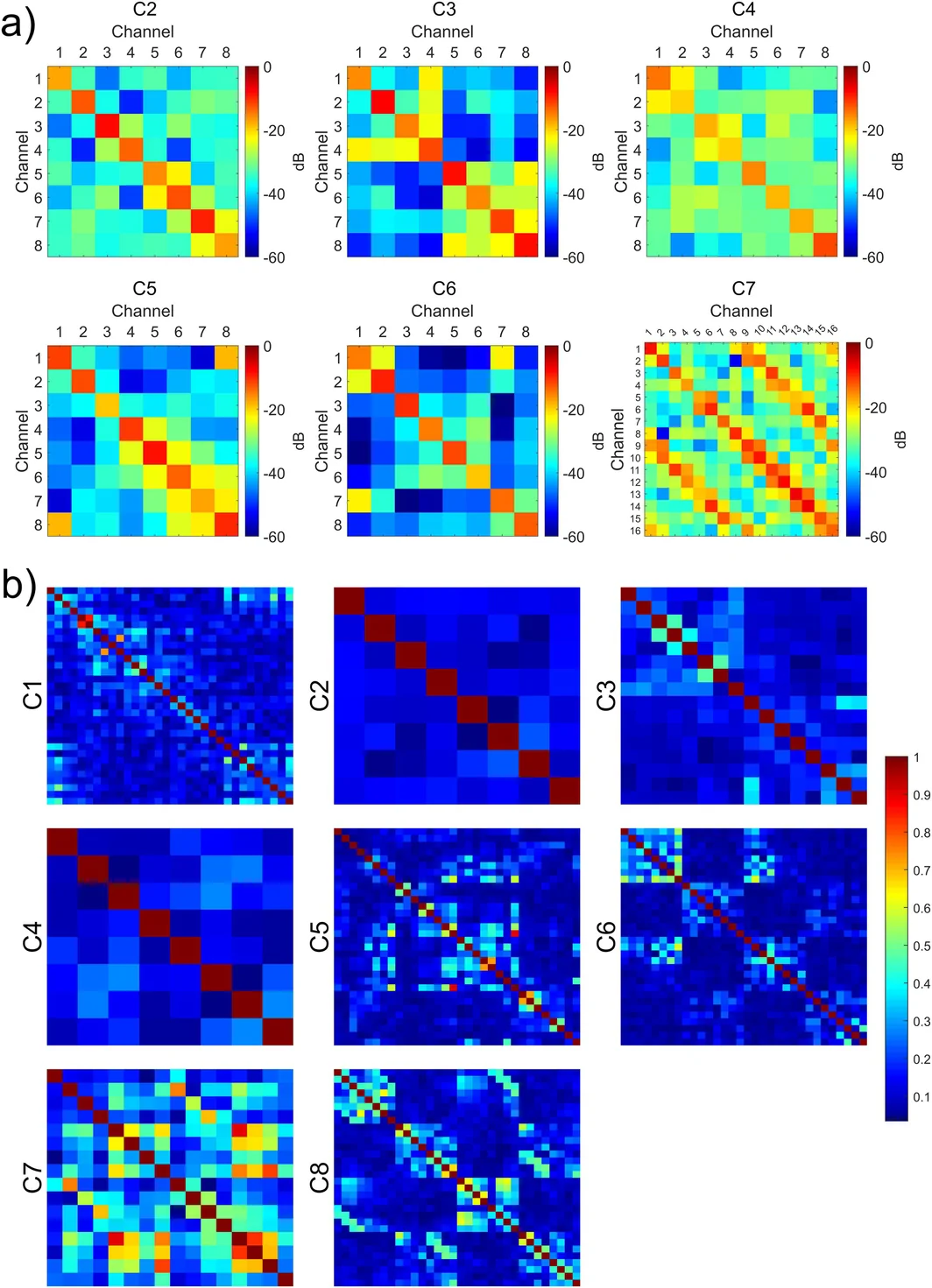
(a) S‐Parameters for all local coils measured on the workbench. For C2, C3, C6 and C7 S‐Parameters were measured after the Tx/Rx switch. For C4 and C5 S‐Parameters were measured without the Tx/Rx switch. C7: Channel 1–8 are dipoles, Channel 9–16 are loops. Average S_ii_ /S_ij_ (i ≠ j) value: C2: −13.3 dB/35.1 dB, C3: −11.8 dB/−37.8 dB, C4: −17.0 dB/−32.2 dB, C5: −12.8 dB/−36.9 dB, C6: −13.4 dB/−43.4 dB, C7 (all channels): −11.1 dB/−28.8 dB, C7 dipoles: −12.6db/−32.1 dB, C7 loops: −9.6 dB/−27.3 dB. (b) Noise correlation matrices for all receive configurations. C1: 32Tx32Rx, C2: 8Tx8Rx, C3: 8Tx16Rx, C4: 8Tx8Rx, C5: 8Tx32Rx, C6: 8Tx32Rx, C7: 8Tx16Rx using dipoles as transmit, C8: 32Rx.

### 3.2 Receive Performance Analysis

#### 3.2.1 SNR

Figure 7 shows the transversal SNR maps for all receive configurations. Among all configurations, the highest central mean SNR was obtained with C5 with 32 receive channels (Figure S5). Figure S6 shows the relative SNR maps of all receive configurations with respect to the remote array C1. All local arrays demonstrated higher SNR than the remote configuration at the phantom center. However, at the left and right edges of the phantom, C3 and C8 exhibited lower SNR compared to the remote array.

**FIGURE 7:**
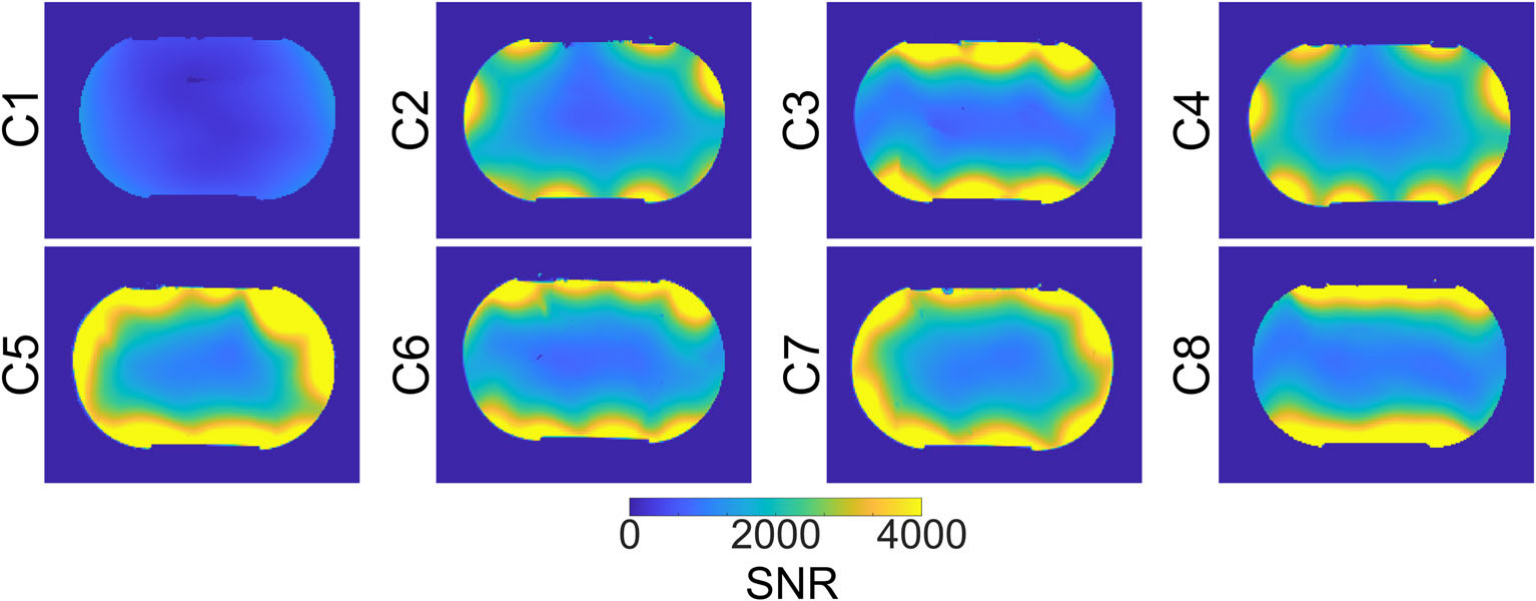
SNR maps for all receive configurations. C1: 32Tx32Rx, C2: 8Tx8Rx, C3: 8Tx16Rx, C4: 8Tx8Rx, C5: 8Tx32Rx, C6: 8Tx32Rx, C7: 8Tx16Rx using dipoles as transmit, C8: 32Rx.

The 8‐receive‐channel configuration (C2) yielded an SNR gain of 176% in the central ROI compared to the remote coil C1, while C4, which also has 8 receive channels but with an upgraded coil housing, achieved an SNR gain of 218% in the center.

The 16‐receive‐channel configuration C3 performed similarly to the 8‐receive‐channel array C4 (214%) in the central ROI, whereas C7 achieved an SNR gain of 284% compared to C1, providing an additional 21%–39% SNR improvement compared to C2/C3/C4 in the center.

Relative to C1, C5, and C8 with 32 receive channels achieved a 285%/281% SNR increase in the center. Despite also employing 32 receive elements, C6 yielded SNR values in the phantom center comparable to the 8‐receive‐channel configurations, achieving an SNR gain of 220% compared to C1.

Figure 8 shows the SNR profile along the HF‐direction through the phantom center, derived from the sagittal SNR maps. The corresponding reception coverage, that is the region in which the SNR of each receive configuration exceeds 75% of its central SNR, is summarized for all receive configurations in the figure caption of Figure 8. The largest coverage was observed for C1, C6, and C8, which extended across the entire FoV, as all three receive configurations include multiple elements distributed along the HF‐direction and were designed for large FoV imaging.

**FIGURE 8:**
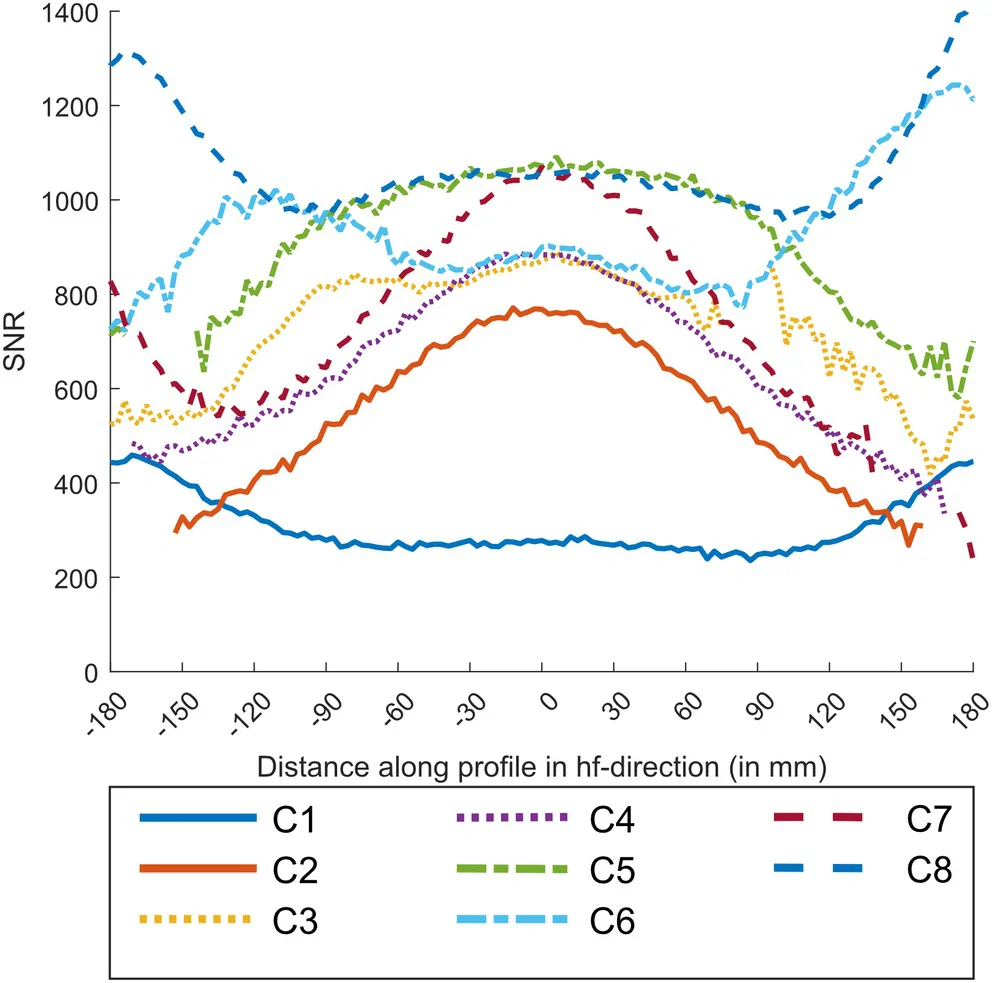
HF‐reception profile for all receive configurations along the isocenter line. The reception coverage of all receive configurations, which is defined as the region where the SNR of each Rx configuration exceeds 75% of its center SNR, is given as follows: C1 = 384 mm, C2 = 141 mm, C3 = 252 mm, C4 = 159 mm, C5 = 246 mm, C6 = 384 mm, C7 = 135 mm, C8 = 384 mm.

The smallest reception coverage was observed for the dipole loop configuration (C7), despite incorporating 16 receive elements, as its elements are confined to a single axial plane receive configurations with a single ring of stripline receive elements (C2, C4) increased coverage by 4%–17% compared with the single‐ring dipole loop configuration (C7).

C6, featuring multiple loop receive elements along the HF‐direction, further improved reception coverage by 58%–87% relative to single‐ring designs (C2, C4, C7). Similar coverage was obtained with C5, although the additional stripline element provided higher SNR in the phantom center.

#### 3.2.2 Noise Correlation

Figure 6b shows the noise correlation matrices for all receive configurations, displaying inter‐channel coupling. All receive configurations exhibited mean noise correlation coefficients below 0.17, showing sufficient element decoupling, except C7, which showed a mean correlation of 0.32 between elements.

#### 3.2.3 Acceleration Potential

Figure 9 shows the g‐factor maps for all receive configurations for acceleration factors (*R* = 2–5) with undersampling in the AP‐ and LR‐direction for the isocenter transverse slice and in HF‐direction for the isocenter coronal slice. The corresponding mean and maximum g‐factor values are given in Figure S7.

**FIGURE 9:**
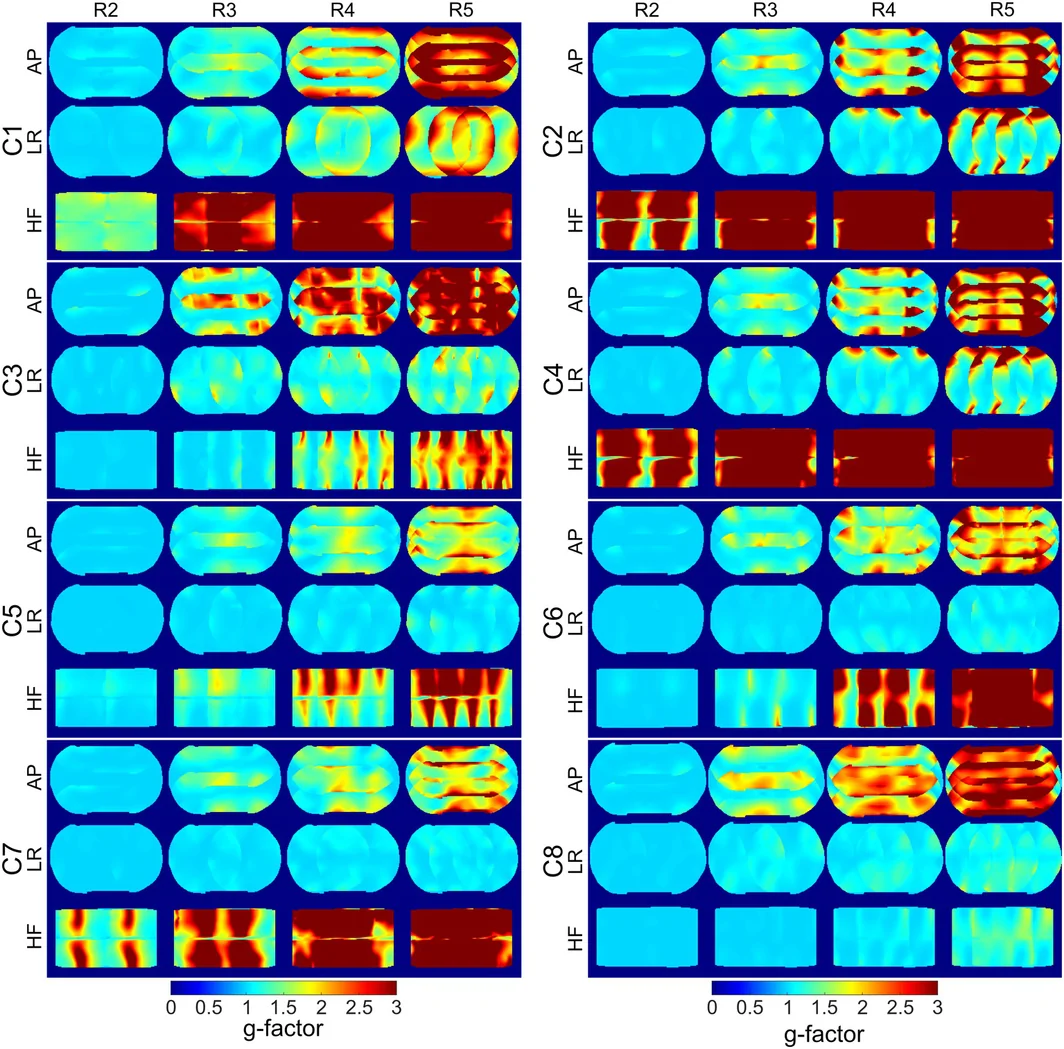
G‐factor maps for acceleration between *R* = 2 and *R* = 5 for all receive configurations using the undersampling patterns of 2D SENSE for acceleration in AP‐direction (top row), LR‐direction (middle row) and HF‐direction (bottom row). C1: 32Tx32Rx, C2: 8Tx8Rx, C3: 8Tx16Rx, C4: 8Tx8Rx, C5: 8Tx32Rx, C6: 8Tx32Rx, C7: 8Tx16Rx using dipoles as transmit, C8: 32Rx.

In general, all receive configurations exhibited lower mean and maximum g‐factor values for acceleration along the LR‐direction than in the AP‐direction. On average, the maximum g‐factors in the AP‐direction were 16%, 51%, 92%, and 241% higher than in the LR‐direction for (*R* = 2, 3, 4, 5), respectively. In HF‐direction, receive configurations with their receive elements placed in a single axial plane (C2, C4, C7) yielded higher g‐factor values than in AP‐direction. Across all receive configurations, maximum g‐factor values ranged from 1.16 to 24.1 in the AP‐direction, 1.01 to 8.71 in the LR‐direction, and 1.02 to 161 in the HF‐direction.

For acceleration in AP‐ and LR‐direction, arrays with 16 or 32 receive channels generally outperformed 8 receive channel arrays. C7 (16 receive channels) achieved g‐factor values comparable to the 32 receive channel arrays (C5, C6) and consistently outperformed C3 (16 receive channels). In contrast, the dedicated 32 receive channel array C8 exhibited slightly higher g‐factors than C7 despite its larger number of receive elements, reflecting its extended element distribution in the HF‐direction.

For acceleration in HF‐direction, distributing receive elements along the HF‐direction substantially reduced g‐factor values. Among these configurations, C8 achieved the lowest mean and maximum g‐factors across all acceleration factors, whereas C7 yielded the lowest g‐factors among arrays with receive elements placed in a single axial plane.

As expected, increasing the number of receive elements reduced g‐factor values and enabled higher acceleration factors in AP‐ and LR‐direction, while distributing the receive elements in HF‐direction enabled higher accelerations in HF‐direction. Among all configurations, C5 consistently achieved the lowest mean and maximum g‐factors across all accelerations in AP‐ and LR‐direction.

## 4 Discussion

In this work, eight RF coils, 7 local arrays and one remote array, assessed in eight unique transmit (C1–C6, C7 dipoles, and C7 loops) and eight receive configurations (C1–C8) were systematically assessed for their respective transmit and receive performances at 7 T. The evaluated coils represented a diverse range of element types, geometries, and channel configurations, reflecting the variety of designs currently employed in UHF body imaging.

Previous review articles by Kraff et al. [20] and Williams et al. [41] provided comprehensive overviews of different 7 T body designs. However, those reviews do not provide a direct, head‐to‐head evaluation of different coils or design strategies.

Although earlier work by Metzger et al. [42] assessed three coil configurations for prostate imaging through simulations and measurements, their inclusion of endorectal coils limits generalizability to broader abdominal targets (e.g., liver or kidneys). Similarly, Rietsch et al. [43] compared single‐element types only as candidates for remote arrays rather than as fully assembled local coils. Furthermore, while most of the coils or element types were already evaluated individually [22, 23, 27, 29], a direct comparison between them has not yet been conducted through phantom measurements. Thus, our comparison of eight transmit and receive configurations represents an important first step toward a more unified performance assessment, even though it still captures only a portion of the existing diversity in 7 T body coils.

To reduce the likelihood that differences originated from external factors rather than the coil designs and configurations, we conducted all measurements using the same MRI scanner. This approach reduced influences from scanner‐dependent hardware variations such as the magnet [44], gradient system [45, 46] or RF amplifiers [5, 47]. In addition, coils C2, C4, C5, and C7 were operated using the same transmit/receive‐switch and preamplifier box. Moreover, a single standardized phantom and unified measurement protocols were employed to achieve a direct comparison of *B*_1_^+^ efficiency, transmit and receive coverage, SNR, noise correlation, and parallel‐imaging performance, as variations in phantom properties or sequence parameters could otherwise introduce variability unrelated to the coil performance itself.

On the transmit side, we observed that transmit configurations with a shorter extension along the HF‐direction typically enabled higher FA within the central ROI, whereas transmit configurations providing wider HF‐coverage did so at the cost of reduced *B*_1_^+^ efficiency. This trade‐off occurs because increased longitudinal coverage distributes the available RF power over a larger region. Moreover, distributing a limited number of elements both circumferentially and along the HF‐direction leads to spatial variation. In practice, such variations can be less suitable, since pTx designs that aim for a homogeneous FA distribution along the HF‐direction need to counteract these intrinsic variations, which is particularly challenging for static pTx, where the degrees of freedom are limited. Thus, from an application perspective, a design with balanced field homogeneity in the HF‐direction seems preferable. Future coil developments may mitigate these limitations through optimized element geometries. Additionally, we observed that using the same element type but in a modified housing with anatomically conformed element positioning in the posterior part is associated with increased transmit efficiency. This observation not only highlights the importance of coil housing design it may also yield to slightly impaired results of coil C7, since this coil required a different housing compared to the original prototype [29] due to regulatory reasons. Furthermore, the transmit evaluation showed that not all coil configurations achieve the conventional reference *B*_1_^+^ level of 11.7 μT that is required for a 180° flip angle with a 1 ms rect‐shaped RF pulse. For these configurations, achieving comparable *B*_1_^+^ amplitudes would require longer RF pulse durations, which may be restrictive for applications requiring short pulses, such as sequences sensitive to *B*_0_ inhomogeneities or those demanding short echo times. Moreover, increasing the pulse duration reduces the RF bandwidth, potentially leading to enhanced off‐resonance effects.

On the receive side, our results show that lateral element placement improves SNR and g‐factor performance, particularly in the AP‐direction. Additionally, increasing the number of receive elements generally enhanced SNR and parallel imaging capabilities across all orientations as expected, although potentially with a minor tradeoff in transmit performance. An additional observation, which is in accordance with experience at lower field strengths, was that a remote transmit coil such as C1 performs poorly when also used for reception, leading to markedly reduced SNR and limiting its usefulness as a standalone transmit/receive solution. This reinforces the need for a dedicated local receive array, as realized by configuration C8, that can provide the necessary proximity to achieve high SNR and high acceleration factors. Consequently, these findings highlight the importance of combining remote transmit arrays with appropriate local receive arrays to exploit the complementary strengths of each design.

This study is also subject to limitations. The study protocol was optimized particularly for the center transversal slice and required approximately 1.5–2 h of measurement time plus calibration. When evaluating the results of the coils, additional specific questions regarding the transmit and receive performance along HF‐direction emerged. To address these questions, we used the GRE‐based raw data that was used to calculate the B1R maps. For the transmit performance evaluation in HF‐direction, the resulting B1R maps were then used since 3D MRF‐based *B*_1_^+^ maps were not available. Although B1R mapping can introduce a bias in *B*_1_^+^ [31], this effect is generally limited and they still provide meaningful and interpretable results. For the receive performance evaluation in HF‐direction, the combined and corrected SNR maps were calculated from the GRE acquisition images used for B1R determination and validated in transversal orientation against the combined and corrected SNR maps derived from the dedicated GRE‐SNR acquisitions. This comparison yielded only a constant SNR offset, which was taken into account.

Additionally, while one set of transmit/receive switches could be used for several configurations, different transmit/receive switches had to be used across coils, as it was not possible to connect all coils to the same switch. This could introduce variations in *B*_1_^+^ efficiency due to different attenuations of the switches. Also, some coils lacked clearly marked geometric centers, resulting in minor positional misalignments that could slightly reduce measured *B*_1_^+^ efficiency. However, since the excitation maxima along the HF‐direction were consistently located near the phantom center across all coils, these errors are expected to be minimal.

In addition, the coils were evaluated using different cable assemblies and positioning. This approach was necessary because each coil was manufactured with its specific cables and we aimed to compare the coils under the conditions in which they would actually be used. Differences in cable length, routing, and shielding can affect the performance of the coils. Although these factors represent potential sources of systematic variability, the setup reflects realistic usage conditions, and care was taken to minimize differences wherever possible.

An additional limitation is that some of the coils evaluated were not originally designed for operation on the non‐conventional 7 T system with an integrated body coil. Differences in system architecture could, in principle, affect coil tuning and impedance matching and thus potentially the coil performance. However, the potential impact on overall coil performance is likely minimal, as the noise correlation matrices of all evaluated coils, with the exception of the C7 coil, indicated sufficient element decoupling.

Additionally, the coil performance was evaluated using a single body phantom. Variations in loading conditions were systematically assessed using S‐parameter measurements acquired both in phantom (Figure 6b) and in vivo on the workbench for the local arrays, with different anatomical loading scenarios created by positioning either the liver (C2, C3, C4, C5, C6, and C7) or the prostate (C2, C3, C6, and C7) at the isocenter (Figure S8). While some differences may depend on subject‐specific loading, the figures showed comparable S‐parameter matrices across the different loading conditions for each coil, with no substantial loading‐dependent variations observed.

A comprehensive comparison of SAR performance, including VOP‐based safety assessment and optimization across different body models, was not performed in this study due to the lack of consistent electromagnetic models for all coil configurations. However, this represents an important and relevant direction for future work.

While this work focused on phase shimming, the observed trends are expected to be relevant for broader pTx applications. The larger coverage in the HF‐direction provided by configurations such as C1 and C6 would likely improve excitation performance for larger fields of excitation when using dynamic pTx approaches. However, intrinsic *B*_1_^+^ variations along the HF‐direction (as observed for C3) would still need to be compensated by dynamic pTx strategies, although dynamic pTx is expected to outperform static phase shimming in mitigating these variations. For smaller FoX, such as the prostate, coil configurations with higher *B*_1_^+^ efficiency are also expected to be advantageous for dynamic pTx applications, as they may facilitate the generation of more efficient RF pulses while maintaining flexibility for SAR‐constrained optimizations. Nevertheless, these considerations were not directly investigated in the present study and remain to be evaluated in future work.

Finally, the g‐factor maps along the HF‐axis were computed using zero‐phase shimming, as no CP+ or efficiency‐shim data were obtained. Thus, in some regions, destructive interference therefore reduced the available information. Nevertheless, these local gaps do not affect the overall trends, and the main conclusions regarding the acceleration performance of the coils remain unaffected.

Beyond recommending specific existing configurations, our results highlight key design principles that might be considered for future coils: (1) decoupling transmit and receive arrays enables independent optimization of transmit performance and coverage and, on the other hand, SNR and SNR coverage, (2) lateral receive element density is critical for acceleration in the AP−/LR‐directions, whereas distributed HF placement is essential for imaging, and (3) element type (dipole vs. loop) strongly influences both shimming efficiency and longitudinal coverage.

## 5 Conclusion

This study offers a valuable overview of the transmit and receive performance of existing pTx coils. A local transmit/receive coil with a high number of receive elements that also covers the sides of the body appears to be a good tradeoff between high SNR and *B*_1_^+^ efficiency. Hereby the transmit element length or element type is selected based on the dimensions of the target volume, ensuring optimal performance. The length of the transmit elements is adapted to the dimensions of the target volume. The remote body coil combined with a high‐channel receive coil requires more total RF power to compensate for its reduced *B*_1_^+^ efficiency. However, it offers more flexibility due to a larger field of excitation, more patient comfort, and easier handling, while providing SNR comparable to that of the local transmit/receive counterpart with the same number of receive elements.

## Funding

This work was supported by Deutsche Forschungsgemeinschaft, SCHM 2677/4‐1; National Institutes of Health, NIBIB P41 EB027061.

## Conflicts of Interest

The authors declare no conflicts of interest.

## Supporting information


**Figure S1:** Details of all coils, showing the individual elements and illustrating their geometry, dimensions, and layout within the array. C3 was adapted from “Accelerated B1+ mapping and robust parallel transmit pulse design for heart and prostate imaging at 7 T” by Egger N et al. available at Magn Reson Med. 2024; 92 (5):1933–1951. doi: 10.1002/mrm.30185, licensed under CC BY 4.0. C5 was adapted from “An 8Tx/32Rx RF Coil for 7 T UHF Body MRI” by Rietsch SHG et al. available at Proceeding of the 16th annual meeting of the ISMRM 2016, Singapore, #2131.
**Figure S2:** Drawing of the body phantom.
**Figure S3:** Scan parameters of the used sequences.
**Figure S4:** Procedure to evaluate the excitation profiles in HF‐direction using C2. (a) SOM(B1R) maps. (b) 2D coronal and sagittal SOM(B1R) maps normalized to the center line marked in red. (c) B1,max+ (green line) is given as the center line of the 2D normalized (green) scaled with mean B1+ in the center ROI, the available input power per channel and Nch. FoX marks the area where B1,max+ (green line) exceeded the 7.8μT (red line).
**Figure S5:** Mean, std. and max SNR values within the circular ROI in the center of the phantom for all Rx configurations. C1: 32Tx32Rx, C2: 8Tx8Rx, C3: 8Tx16Rx, C4: 8Tx8Rx, C5: 8Tx32Rx, C6: 8Tx32Rx, C7: 8Tx16Rx using dipoles as Tx, C8: 32 Rx.
**Figure S6:** Relative SNR maps for all Rx configurations with respect to C1. C1: 32Tx32Rx, C2: 8Tx8Rx, C3: 8Tx16Rx, C4: 8Tx8Rx, C5: 8Tx32Rx, C6: 8Tx32Rx, C7: 8Tx16Rx using dipoles as Tx, C8: 32Rx.
**Figure S7:** G‐factor values for all Rx configurations and for acceleration factors between R = 2 and R = 5 in AP‐, LR‐ and HF‐direction. C1: 32Tx32Rx, C2: 8Tx8Rx, C3: 8Tx16Rx, C4: 8Tx8Rx, C5: 8Tx32Rx, C6: 8Tx32Rx, C7: 8Tx16Rx using dipoles as Tx, C8: 32Rx.
**Figure S8:** S‐Parameter matrices of all local coils measured on the workbench in vivo. For C2, C3, C6 and C7 S‐Parameters were measured after the Tx/Rx switch. For C4 and C5 S‐Parameters were measured without the Tx/Rx switch. C7: Channel 1–8 are dipoles, Channel 9–16 are loops.

## Data Availability

The measurement data of all coils will be provided upon reasonable request.
