# Supplementary material for: Evaluation of 7 Tesla pTx Body Coils Regarding Transmit and Receive Performance
Source: Magn Reson Med. 2026 Aug 14;96(6):2988–3001. doi: 10.1002/mrm.70557 (PMC13619844; doi:10.1002/mrm.70557)
Supplement: Supplementary file 1 [file MRM-96-2988-s001.docx]

Figure S1: Details of all coils, showing the individual elements and illustrating their geometry, dimensions, and layout within the array. C3 was adapted from “Accelerated B1+ mapping and robust parallel transmit pulse design for heart and prostate imaging at 7 T” by Egger N et al. available at Magn Reson Med. 2024;92(5):1933-1951. doi:10.1002/mrm.30185, licensed under CC BY 4.0. C5 was adapted from “An 8Tx/32Rx RF Coil for 7T UHF Body MRI” by Rietsch SHG et al. available at Proceeding of the 16^th^ annual meeting of the ISMRM 2016, Singapore, #2131.

Figure S2: Drawing of the body phantom.

Figure S3: Scan parameters of the used sequences.

Figure S4: Procedure to evaluate the excitation profiles in HF-direction using C2. a) SOM(B1R) maps. b) 2D coronal and sagittal SOM(B1R) maps normalized to the center line marked in red. c) B_1,max_^+^ (green line) is given as the center line of the 2D normalized (green) scaled with mean B_1_^+^ in the center ROI, the available input power per channel and N_ch_. FoX marks the area where B_1,max_^+^ (green line) exceeded the 7.8µT (red line).

Figure S5: Mean, std and max SNR values within the circular ROI in the center of the phantom for all Rx configurations. C1: 32Tx32Rx, C2: 8Tx8Rx, C3: 8Tx16Rx, C4: 8Tx8Rx, C5: 8Tx32Rx, C6: 8Tx32Rx, C7: 8Tx16Rx using dipoles as Tx, C8: 32 Rx.

Figure S6: Relative SNR maps for all Rx configurations with respect to C1. C1: 32Tx32Rx, C2: 8Tx8Rx, C3: 8Tx16Rx, C4: 8Tx8Rx, C5: 8Tx32Rx, C6: 8Tx32Rx, C7: 8Tx16Rx using dipoles as Tx, C8: 32Rx.

Figure S7: G-factor values for all Rx configurations and for acceleration factors between R=2 and R=5 in AP-, LR- and HF-direction. C1: 32Tx32Rx, C2: 8Tx8Rx, C3: 8Tx16Rx, C4: 8Tx8Rx, C5: 8Tx32Rx, C6: 8Tx32Rx, C7: 8Tx16Rx using dipoles as Tx, C8: 32Rx.

Figure S8: S-Parameter matrices of all local coils measured on the workbench in vivo. For C2, C3, C6 and C7 S-Parameters were measured after the Tx/Rx switch. For C4 and C5 S-Parameters were measured without the Tx/Rx switch. C7: Channel 1-8 are dipoles, Channel 9-16 are loops.
